# Immersive Virtual Learning Environments for Healthcare Education: State-of-Art and Open Problems

**DOI:** 10.7759/cureus.68483

**Published:** 2024-09-02

**Authors:** Bill Kapralos

**Affiliations:** 1 maxSIMhealth Group, Ontario Tech University, Oshawa, CAN

**Keywords:** augmented reality, virtual reality, healthcare education, serious game, virtual simulation, immersive virtual learning environment (ivle)

## Abstract

Immersive virtual learning environments (iVLEs), serious games and virtual simulations in particular, allow trainees to learn from virtual simulated experiences in an interactive, engaging, and ethically safe manner. Although the use of iVLEs in healthcare education was steadily growing over the last 10-15 years, their use was accelerated during the coronavirus disease 2019 (COVID-19) pandemic as they played a significant role in facilitating remote learning during the pandemic-related lock-downs. Within healthcare education, iVLEs are not so much technologies of the future but rather, of the present. Educators are finding innovative ways to leverage the coupling of iVLEs and emerging technologies such as extended reality (augmented, mixed, and virtual realities), and artificial intelligence (AI), to transform healthcare education. Despite the growing popularity and benefits of iVLEs, there are several issues/limitations in their current form including the following three that particularly limit their use. More specifically, iVLEs typically i) contain fixed/static scenarios leading to predictability, disengagement, and repetition in learning experiences, ii) emphasize cognitive skills in contrast to psychomotor skills given the difficulties and cost associated in simulating the sense of touch which is inherent in psychomotor-based tasks, and iii) are not inclusive and therefore, do not properly address user diversity and accessibility. With a focus on healthcare education, this article begins with an overview of iVLEs, followed by a discussion regarding these limitations and potential solutions to overcome these limitations.

## Editorial

Introduction

An immersive virtual learning environment (iVLE) is a computer-generated, three-dimensional (3D) space where trainees can interact with virtual objects and scenarios as if they were real. For example, in medical education an iVLE can simulate an operating room where trainees can practice complex surgical procedures without any risk to actual patients. Trainees can wear a head-mounted display and haptic gloves allowing them to manipulate virtual surgical tools in a 3D space providing them resistance as they make incisions or suture wounds. iVLEs and more specifically, serious games (video games designed specifically for educational purposes), and virtual simulations, shifted from a “backburner training tool to a first-choice strategy for ensuring individual, team, and system readiness”, playing an integral part in facilitating online learning during the abrupt move to remote (virtual) learning given coronavirus disease 2019 (COVID-19) pandemic lockdowns/stay-at-home orders [1]. For example, the Colorado State University implemented an eight-week virtual reality (VR) course to supplement online human anatomy instruction. Seventy-five trainees were provided with a laptop and head-mounted display and participated in weekly synchronous group laboratory sessions that included working collaboratively with other students and instructors in a common virtual space with a virtual cadaver [2]. There are many benefits to remote learning and the use of iVLEs, including the removal of geographical boundaries, flexibility (for the educators and trainees), and it is a more environmentally friendly option (e.g., reduced driving to/from schools). The popularity of remote learning and iVLEs has increased tremendously since the pandemic and is expected to grow further post-pandemic.

Despite the growing popularity and benefits of iVLEs, there are various issues/limitations in their current form, particularly the following three which can limit their application and prevent more widespread use. More specifically, the majority of iVLEs typically: i) contain fixed/static scenarios leading to predictability, disengagement, and repetition in learning experiences which, after several sessions, can limit their effectiveness as teaching tools [3], ii) focus on cognitive skills development (the development of the mental skills and the acquisition of knowledge), and do not address psychomotor skills development (psychomotor skills development integrates both mental and muscular activity to learn motor skills [4]); the sense of touch is inherent in psychomotor skills development, yet simulating the sense of touch in the virtual domain is difficult and expensive, and iii) are not inclusive since they do not address user diversity and accessibility; this is particularly the case for visually impaired users who encounter greater accessibility barriers given the inherent interactive and visual nature of most iVLEs [5].

For the remainder of this editorial, a discussion regarding solutions to the three problems previously discussed (fixed scenarios, lack of psychomotor skills development, and lack of inclusivity) is provided followed by a brief discussion of current ongoing work taking place within the maxSIMhealth Lab at Ontario Tech University in Oshawa, Canada that brings together the individual solutions to these problems in a novel “no-code” anesthesia crisis scenario authoring platform (ACSAP). The ACSAP will allow educators with limited (if any) programming experience to develop new (or modify existing) inclusive iVLEs, specific to anesthesia training, that incorporate pseudo-haptics. As described below, pseudo-haptics refers to the use of audio, visual, and/or kinesthetic cues to create haptic sensations which can be used to create the illusion of touch and thus allow for (limited) psychomotor skills development in iVLEs.

iVLE authoring platforms

**Figure 1:**
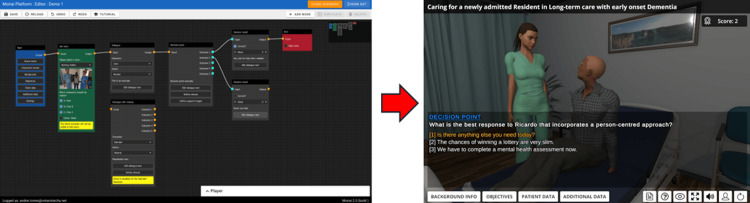
Sample screenshot of the diagram-based user interface of the Moirai authoring platform (left), and sample screenshot of the resulting virtual simulation (right). [12]

There is a growing need for tools that empower educators, even those with limited (if any) programming knowledge to create customized educational content easily and rapidly, avoiding the limitations of fixed scenarios and one-size-fits-all approaches inherent with most iVLEs. Such an approach requires new development methods (“authoring platforms”) that allow educators who may have limited (if any) programming/technical knowledge to easily create/modify iVLEs [6]. Although not specific to iVLEs, large technology companies such as IBM, Microsoft, and Amazon are investing in empowering end users to create applications for their platforms applying a “low-code” or “no-code” approach that replaces (either partially or completely, respectively) programming with visual tools to develop software [7]. With respect to iVLEs, although not widespread, some prior work has examined low-code authoring platforms. Chabbi et al. proposed a game software architecture for configurable serious games, allowing creators to define parameters and build platform-independent components [8]. The Learning Inter-Professionally Healthcare Accelerator (LIPHA) is an educational platform where using self-authoring and self-serve dashboards, educators can tailor content, language, and materials within a virtual simulation or serious game to their target trainees [9]. Although LIPHA can be tailored for healthcare professionals and trainees across any healthcare sector and across any clinical population, currently it is being used to train care providers and long-term care nurses in Ontario, Canada [9]. The work taking place within the maxSIMhealth Lab has seen the development of two no-code healthcare-based iVLE authoring platforms that cater to educators with limited programming experience. The first platform, the Anesthesia Crisis Scenario Builder (ACSB), provides healthcare educators the opportunity to develop new (or modify existing) anesthesia-based virtual simulations that follow the steps found in the Anesthetic Crisis Manual (ACRM) [10]. The ACRM is a collection of 22 life-threatening crises that anesthetists manage in their everyday practice [11]. Once a simulation is created, the ACSB framework generates a virtual operating room and sets up all the educator-specified interactions for trainees to practice. The second platform, called Moirai, empowers educators to easily create new (or modify existing) serious games focused on decision and communication skills development, using a diagram-based interface [12]. Figure 1 provides a sample screenshot of the diagram-based user interface of the Moirai authoring platform (left), and a sample screenshot of the resulting serious game (right).

Pseudo-haptics

Psychomotor skills development in iVLEs is typically ignored since simulating the sense of touch, inherent in psychomotor skills development, is difficult and costly, requiring the use of high-fidelity haptic devices that are not available at the consumer level [13]. Pseudo-haptics refers to the illusion of haptic sensations using combinations of audio, visual, and kinesthetic cues without the use of haptic interfaces [14]. For example, in a virtual simulation for dental implant training, the trainee can control the virtual drill using a standard computer mouse. Pressing one of the mouse keys activates the drill while moving the mouse forward/backward moves the drill in/out of the patient's jawbone. The speed of the cursor varies depending on the material that the drill encounters (e.g., the cursor slows down when the drill encounters denser material), and appropriate sound effects (e.g., drill sounds) are included. This visual and auditory feedback tricks the brain into perceiving resistance, mimicking the feel of actual drilling. Pseudo-haptics has been employed to convey various touch-based properties including to identify tumors during palpation [15]. A thorough review of pseudo-haptics including a discussion regarding the use of pseudo-haptics in training, assistance, and entertainment applications is provided by Ujitoko and Ban [14].

The maxSIMhealth Lab recently began investigating pseudo-haptics as a means of providing a cost-effective method for healthcare-based psychomotor skills development in a remote (online) setting. This included the completion of a study that examined whether pseudo-haptics could simulate a drilling task (common in various healthcare procedures) using standard computer hardware and more specifically, a basic 2D mouse, headphones, and a monitor (2D display) [16]. Participants were tasked with drilling to a specific length (15 cm) through a virtual piece of wood in a first-person perspective. They controlled a virtual drill using a standard 2D computer mouse where the left button activated the drill, moving the mouse forward/backward moved the drill toward/away from a block of wood, and releasing the left button caused the drill to stop. The results of this study indicate that audio-visual cues can provide a suitable perceptual drilling experience without a haptic device [16]. More recently, the maxSIMhealth Lab examined the effect of coupling embodiment (where a virtual hand representing the user’s hand in a first-person perspective held the drill) and pseudo-haptics on performance. Embodiment is fundamental to psychomotor skills development; one cannot acquire any new skills without being bodily present to generate, control, and exploit movement [17]. Results of this study revealed that the addition of a virtual hand significantly enhances the speed of acquisition of a psychomotor skill [18].

Inclusive design and iVLEs

Inclusive design is concerned with enabling people with varying backgrounds and abilities including age, culture, economics, education, race and accessibility with the “focus on fulfilling as many user needs as possible, not just as many users as possible” [19]. There has been very little effort placed on addressing the inclusivity of iVLEs, and access for diverse trainees, particularly those with disabilities who may require accommodations to use them. With respect to iVLEs and video games in general, inclusivity can be divided into two components: i) accessibility, and ii) diversity; both of which involve acknowledging the existence of people and/or groups who lack social power, prestige, or entrenched advantage, and establishing an awareness of their marginalization or exclusion based on social or physical barriers [20].

Accessibility represents the features that developers design into iVLEs and video games to allow access and use of iVLEs/video games by users with a wide range of needs [21]. The Game Accessibility Guidelines (GAGs) were created by a collaboration between a group of game studios, specialists, and academics [22]. The GAGs are divided into three categories: i) basic, ii) intermediate. and iii) advanced, each of which is further subdivided into sub-categories that relate to types of disabilities: 1) motor, ii) cognitive, iii) vision, iv) hearing, v) speech, and vi) general. In Canada, the Web Content Accessibility Guidelines (WCAGs) are used to ensure that web content is inclusive in design [23]. Westin et al. recommend that the WCAGs and GAGs are used in conjunction in iVLEs/video games [24]. Recent work involved the application of the GAGs and WCAGs to develop an accessible version of Foodbot Factory, a serious game to teach adolescents about nutrition and Canada’s Food Guide [25]. This work also led to a set of recommendations for converting a non-accessible serious game into an accessible version [25].

According to the non-profit International Game Developers Association (IGDA), diversity is any dimension that can be used to differentiate groups and people from one another and may include culture, race, ethnicity, physical appearance, age, gender identity, sexual orientation, ability/disability, neurodiversity, socioeconomic, behaviour, ethno-diversity, ideologies and viewpoints, education, career and roles, marital/parental status, location and geography, history, technology, and its access [26]. Armstrong conducted a review that examined over 160 studies on representation in media, and reports a predominance of white characters in software, books and learning tools and a significant lack of representation of people of colour [27]. Various inclusivity guidelines have been proposed for games and media in general. For example, the IGDA has devised the “Inclusive Game Design and Development” framework that emphasizes diversity for designing and developing inclusive games and media and provides a series of steps and considerations for every stage of the design and development process to help create inclusive games as well as empower underrepresented players and create an impassioned fan-base [26].

Putting it all together

Work will begin within the maxSIMhealth Lab that will see the development of the novel (and freely available) “no-code” ACSAP that builds upon the previously described ACSB and Moirai authoring platforms. The ACSAP will allow educators with limited (if any) programming experience to easily develop new (or modify existing) iVLEs for anesthesia training using a drag-and-drop approach without any programming using a scenario editor. The iVLEs created with the ACSAP will conform to accessibility standards (visual, hearing, motor, and cognitive), and will ensure diversity by following the IGDA framework that includes incorporating customization tools allowing the trainees using the iVLEs to individualize their avatars (the digital representation of themselves) in the iVLE (e.g., skin colour, hair, clothing, voice), and other aspects of the environment. Through the integration of our novel work in pseudo-haptics, the ACSAP will remove the need for expensive haptic devices and allow for psychomotor skills (e.g., “hands-on”) development using standard computer hardware in a remote setting. The ACSAP will focus on “needle and syringe” type procedures (e.g., intravenous (IV) placements, and the epidural procedure in particular), taking advantage of our prior work that has included development of iVLEs for cognitive skills learning for these procedures (see Kapralos et al. [13]). The completion of a fully functioning prototype is expected within the next 12-18 months at which point, formal effectiveness testing will take place where both the scenario editor and the resulting iVLEs created using the scenario editor will be tested. The aim is to make the ACSAP freely available along with a common repository where users can share any scenarios they create amongst each other.

Limitations

Despite the potential benefits of the ACSAP, it should be noted that there are several limitations. Although pseudo-haptics will be used to provide haptic sensations and thus allow for psychomotor skills development, pseudo-haptics is limited and cannot fully simulate the sense of touch required for high-fidelity psychomotor skills development. The intent is to provide a portable and cost-effective "middle-ground" between iVLEs that focus solely on cognitive skills development and those that offer full psychomotor skills development with high-fidelity haptic devices housed in simulation laboratories. Furthermore, developing any authoring platform aimed at users (educators) with limited technical knowledge is challenging. More specifically, determining the threshold of the appropriate trade-off between functionality and ease of use and determining how much time educators are willing to devote to learning how to use such a platform are both difficult to answer. A survey to gauge educators’ technical skill set and knowledge, their willingness to develop interactive software and games, given authoring platforms, and what features they would like to see in such platforms could be developed and distributed. 

Aside from the issues focused on here, it should be noted that there are additional issues with iVLEs that require addressing including the lack of lack personalization and therefore failure to account for individual learning needs. Here, artificial intelligence (AI) can be helpful. In particular, AI can be used to provide an adaptive iVLE where an automatic adjustment process of the simulation components and difficulty levels, among other factors, is made depending on the learning needs of each trainee. In addition, AI-based virtual assistants (chatbots) can help facilitate adaptation (personalization) by, amongst other things, providing the trainee with a personal tutor that can assist them throughout the learning process depending on their needs. The lack of guidelines/standards to properly integrate iVLEs into the curriculum, and the question regarding how realistic iVLE scenarios must also be addressed. Finally, despite the increase in computer power available to the average computer user, rendering highly realistic, interactive iVLE scenarios can still be problematic and out of reach to the average trainee.

Concluding remarks

The availability of cost-effective authoring platforms that allow for psychomotor skills development in a remote setting without the need for expensive hardware devices, and address inclusivity, will remove the primary barriers inherent in iVLEs and empower educators to meet the diverse needs of trainees. Here, ongoing work within the maxSIMhealth Lab that is seeing the development of the ACSAP authoring platform that addresses these issues by allowing educators to easily create new (or modify existing) iVLEs specific to anesthesia training with limited (if any) programming experience was introduced.
